# TRIM proteins: New players in virus-induced autophagy

**DOI:** 10.1371/journal.ppat.1006787

**Published:** 2018-02-01

**Authors:** Konstantin M. J. Sparrer, Michaela U. Gack

**Affiliations:** Department of Microbiology, University of Chicago, Chicago, Illinois, United States of America; University of Kentucky, UNITED STATES

## Role of autophagy during viral infection

Autophagy is an evolutionarily conserved and intricately regulated cellular process in which damaged or aggregated proteins, organelles, and pathogen-derived components are engulfed by double-membrane structures, termed autophagosomes, and targeted for lysosomal degradation [1]. The autophagic process consists of distinct phases that include nucleation, autophagosome formation, selection of cargo, autophagolysosomal fusion, and cargo degradation. Autophagy induction is governed by a set of kinases, including Unc-51-like autophagy activating kinase 1 (ULK1), that activate autophagy via assembly of an essential Beclin-1-containing complex. Conversely, the mammalian target of rapamycin (mTOR) is an important negative regulator of autophagy initiation. A hallmark of autophagic flux is the conversion of the microtubule-associated protein light chain 3B (LC3B) from a cytosolic form to a phosphatidylethanolamine-lipidated, membrane-associated form decorating autophagosomes in punctae-like structures. Several forms of autophagy have been identified, including starvation-triggered autophagy, which is a nonspecific autodigestive response, and selective autophagy, in which “tagged” cargos are specifically recognized by sequestosome1 (SQSTM1)-like receptors, such as p62/SQSTM1 or nuclear dot protein 52 (NDP52) [2]. As such, autophagy has been implicated in multiple cellular processes, including stress adaptation, protection against inflammation, neurodegeneration, and antimicrobial activities [3].

Autophagy is induced upon infection by many different viruses from diverse families, yet the impact of the autophagic host response on viral replication is highly virus and cell-type specific [3]. Some viruses or their components are degraded by autophagy, which limits virus replication and thus serves as an antiviral defense pathway. Additionally, autophagosomes can capture viral components and expose pathogen-associated molecular patterns to innate immune sensors (e.g., Toll-like receptors) upon fusion with endosomes, ultimately promoting innate immune recognition and cytokine-mediated host defenses [4]. Among the viruses sensitive to autophagy-mediated clearance are herpes simplex virus type 1 (HSV-1), Sindbis virus (SINV), and human immunodeficiency virus type 1 (HIV-1). On the other hand, viruses like encephalomyocarditis virus (EMCV), dengue virus, and Zika virus subvert autophagy to promote their replication, and inhibition of autophagic flux suppresses the replication of these viruses [5]. Given the important antiviral and proviral roles of autophagy, it is not surprising that many viruses are equipped with sophisticated mechanisms to modulate autophagy in the infected host cell. For example, EMCV induces autophagy through ER stress via its nonstructural proteins 2C and 3D [6], whereas the γ34.5 protein of HSV-1 suppresses autophagy by interacting with Beclin-1 [7]. Furthermore, the M2 protein of influenza A virus (IAV) modulates autophagy by sequestering LC3B to nonautophagosomal membranes, thereby facilitating virion stability [8].

## Emerging role of TRIM proteins in virus-induced autophagy

A major family of antiviral molecules with approximately 80 members in humans are tripartite motif (TRIM) proteins, which are characterized by the presence of an N-terminal Really Interesting New Gene (RING) domain, one or two B-boxes, and a coiled-coil domain [9]. While most TRIM proteins encode ubiquitin E3 ligase activity conferred by the RING domain, distinct C-terminal domains allow for specific protein—protein interactions or harbor additional enzymatic activities. Whereas TRIM proteins are widely recognized as important antiviral restriction factors or modulators of signaling pathways leading to the induction of antiviral or proinflammatory cytokines, such as type I interferons (IFNs), their roles in other cellular pathways are less well established [9].

A recent series of studies showed that autophagy and antiviral cytokine responses are intricately interconnected [3,4]. Several key molecules implicated in IFN induction are also important regulators of the autophagy pathway. For example, autophagy-related 5 (Atg5)-Atg12 and the nucleotide-binding oligomerization domain (NOD)-like receptor family member X1 (NLRX1) dually regulate virus-induced type I IFN production and autophagy [10,11]. Furthermore, the immunoregulatory kinase TANK-binding kinase 1 (TBK1), which is well known to regulate IFN induction through phosphorylation of IFN regulatory factor 3 and 7 (IRF3/7), also promotes autophagic clearance by phosphorylating the autophagy receptor p62 [12–14].

Interestingly, recent studies demonstrated that several TRIM proteins are key regulators of both viral- and non-viral-induced autophagy, further supporting the intricate intertwining of IFN-mediated innate immunity and autophagy. In a cDNA screen, 31 out of 61 tested TRIM proteins triggered green fluorescent protein (GFP)-LC3B puncta formation, indicative of autophagy induction [14]. An independent study measuring the effect of small interfering RNA (siRNA)-mediated depletion of TRIM proteins showed that autophagy triggered by treatment with pp242 (an mTOR inhibitor) was dependent on 21 TRIM proteins [15]. Another study found 24 TRIM proteins to be essential for IFNγ-induced autophagy [16]. Moreover, a targeted TRIM RNAi screen examining the effect of silencing individual TRIMs on autophagy induction by either EMCV, IAV, or HSV-1 infection, revealed that several TRIM proteins regulate autophagy induced by a particular virus, for example, only during infection with HSV-1 but not IAV or EMCV [14]. Intriguingly, TRIM proteins regulating innate immune pathways triggered by specific viruses (e.g., TRIM25 activating the viral RNA sensor retinoic acid-inducible gene-I (RIG-I) and thereby IFN induction in response to IAV; TRIM56 regulating stimulator of interferon genes (STING) signaling in response to HSV-1 infection) were also essential for autophagy induction by those same viruses. This suggests that the role of these TRIM proteins in autophagy during viral infection is tightly interconnected with their functions in IFN-mediated antiviral immunity. Moreover, some TRIM proteins (e.g., TRIM21, TRIM23, and TRIM41) were found to be required for autophagy induction by several viruses [14], suggesting that these TRIM proteins are core components of the autophagy machinery, or act at a common step of the autophagy induction pathway.

**Fig 1:**
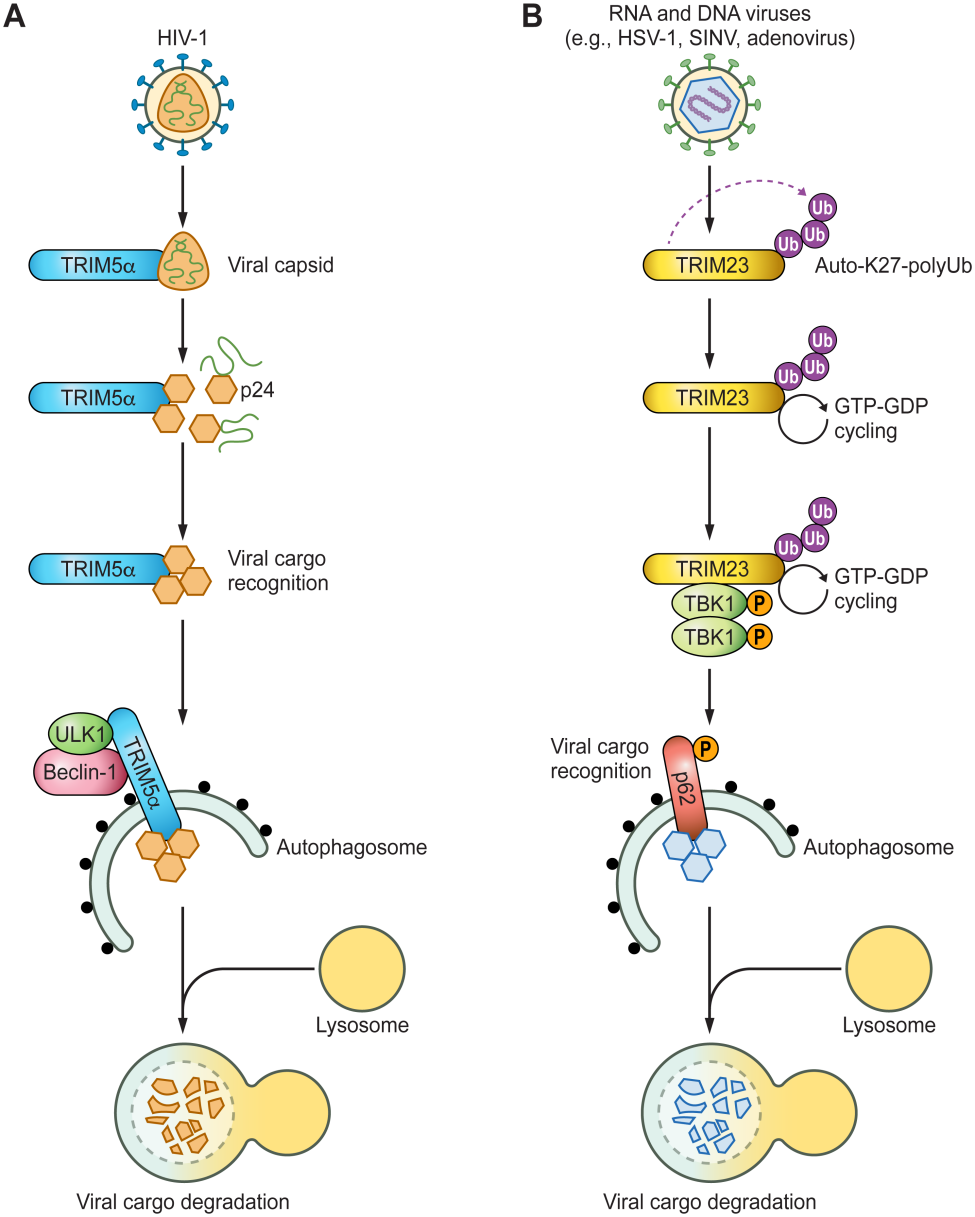
TRIM proteins modulate virus-induced autophagy. TRIM proteins have at least two different modes of action during virus-induced autophagy. They can act as autophagy receptors that specifically recognize and target viral components for autophagolysosomal degradation (exemplified here by TRIM5α **[A]**), or they regulate the activity of key autophagy molecules (exemplified here by TRIM23 **[B]**). **(A)** After infection of a cell with HIV-1, TRIM5α binds to the capsid of HIV-1, inducing premature capsid disassembly and virus restriction. Both proteasomal (not illustrated) and autophagosomal degradative mechanisms reportedly play a role in viral capsid protein (p24) degradation. For autophagic clearance, the p24-TRIM5α complex is recruited to nucleated autophagosomes, where TRIM5α induces autophagy in a Beclin-1- and ULK1-dependent manner. **(B)** Upon infection by diverse viral pathogens (e.g., HSV-1, SINV, adenovirus), the E3 ligase activity of TRIM23 is induced, which leads to atypical K27-linked auto-polyubiquitination of its C-terminal ARF domain. This modification triggers GTP-GDP cycling of the TRIM23 ARF GTPase, which, upon binding of TRIM23 to TBK1, induces dimerization and trans-autophosphorylation of TBK1 and, thereby, activation of the kinase. Activated TBK1 then phosphorylates the autophagy receptor p62 at specific serine residues, which allows p62 to recognize specific cargos, such as viral proteins, ultimately triggering their degradation via the lysosome. ARF, ADP ribosylation factor; Auto-K27-polyUb, auto-K27-linked polyubiquitination; E3, E3 ubiquitin ligase; GTP-GDP, guanosine triphosphate-guanosine diphosphate; HSV-1, herpes simplex virus type 1; P, phosphorylation; SINV, Sindbis virus; TBK1, TANK-binding kinase 1; TRIM, tripartite motif; ULK1, Unc-51-like autophagy activating kinase 1; Ub, ubiquitin.

At least two different mechanisms of action have been proposed for TRIM proteins acting in antiviral autophagy. Some TRIM proteins act as specific cargo receptors that directly recognize viral components and target them for degradation by autophagy. Other TRIM proteins regulate the activity of key signaling proteins involved in different steps in the autophagy pathway (Fig 1).

## TRIM proteins acting in cargo recognition

The concept of TRIM proteins acting as “autophagy receptors” by recognizing viral proteins as cargo has been most well characterized for TRIM5α, which was initially discovered as an antiviral restriction factor that recognizes the HIV capsid protein [17]. While Old World monkey TRIM5α is able to recognize HIV-1 capsids, the human TRIM5α fails to do so. Human TRIM5α, however, still retains the ability to bind to certain murine retroviral capsids, demonstrating species-specific restriction. Several different mechanisms have been proposed for TRIM5α-mediated retroviral restriction [18]. Recognition of the HIV-1 capsid by the SPRY domain of TRIM5α leads to premature capsid disassembly and degradation. Capsid degradation was thought to be mediated by the proteasome, but more recently, TRIM5α has been reported to initiate autophagy through recruitment of two key components of the autophagy machinery—ULK1 and Beclin-1—thereby eliciting autophagy-dependent degradation of the HIV capsid [15] (Fig 1). It has been proposed that formation of the TRIM5α lattice around the capsid may trigger autophagy-mediated restriction of HIV-1 infection [19]. However, other data suggested that retroviral restriction by TRIM5α can still be observed in systems lacking key autophagic components [20], implying that autophagy is not the sole contributor to TRIM5α’s restrictive phenotype and that nondegradative mechanisms likely contribute to retrovirus restriction by TRIM5α. A recent screen showed that silencing of TRIM5α also abrogated autophagy induction by HSV-1 infection [14], suggesting that the role of TRIM5α during autophagy is not limited to retroviral infection. Future studies will need to establish the role of TRIM5α in autophagy during infection by other viral pathogens. Furthermore, the relative contribution of autophagy as compared to other degradative pathways to TRIM5α-mediated retroviral restriction will require further investigation. Finally, although a role for several other TRIM proteins, such as TRIM20 and TRIM21, in autophagy cargo recognition has been reported [16], the relevance of those TRIM proteins in virus-induced autophagy remains to be determined, opening up exciting new avenues of research in virology.

## TRIM proteins functioning as regulators of the autophagy machinery

Independent screening approaches identified several TRIM proteins that play essential roles in both nonviral and viral-induced autophagy, suggesting a key role for these TRIMs in the autophagic process [14–16]. The precise molecular mechanism for most of these TRIMs has not yet been elucidated; however, recently, the role of TRIM23 during virus-induced autophagy was characterized in detail. Silencing or gene targeting of TRIM23 abrogated autophagic flux triggered by a broad range of viral pathogens, which correlated with increased replication of the autophagy-sensitive viruses SINV, adenovirus, and HSV-1, suggesting that TRIM23 mediates virus clearance by autophagy [14]. TRIM23 is unique among TRIM proteins in that it exhibits two enzymatic functions: E3 ubiquitin ligase activity in the RING domain, as most TRIM proteins, and GTPase activity at its C-terminal ARF domain. Thus, TRIM23 can be considered as a fusion protein of a classical tripartite motif and a member of the ARF protein family, which are small (about 20 kDa) GTPases known to modulate cellular membranous processes. Interestingly, the enzymatic activities of TRIM23 are intricately connected and both are required for autophagy induction. Using its RING E3 ligase activity, TRIM23 auto-ubiquitinates its ARF domain, and this posttranslational modification is necessary for TRIM23-mediated autophagic flux and antiviral activity. In-depth biochemical characterization revealed that the ARF domain of TRIM23 is modified by atypical K27-linked polyubiquitin chains, which activate the cycling activity of its GTPase (Fig 1). Mechanistic studies showed that GTP to GDP cycling of TRIM23 facilitates TBK1 dimerization and trans-autophosphorylation and, thereby, activation of the kinase. TBK1 then proceeds to phosphorylate p62 to induce selective autophagy, ultimately promoting viral clearance [14]. These results indicated that the TRIM23-TBK1-p62 axis is important for autophagy-mediated antiviral defense. As both TRIM23 and TBK1 play prominent roles also in the antiviral IFN response, future studies will need to determine the relative contribution of their autophagy- and IFN-regulatory functions to virus restriction. Furthermore, the impact of TRIM23 on the replication of viruses known to usurp autophagy for their efficient replication warrants further investigation.

## Concluding remarks and perspectives

The recent discovery of the role of TRIM proteins in viral autophagy opens up an entirely new area of research in antiviral intrinsic immunity. Whereas the mechanism of autophagy regulation is unknown for most TRIM proteins, it is becoming clear that some TRIM proteins act as receptors recognizing viral molecules, while others are critical components of the autophagy machinery that mediates virus clearance. Although most viruses induce autophagy, the viral signals that trigger autophagy as well as the upstream signaling pathways initiated upon virus-induced autophagy are not very well defined yet. Furthermore, as key autophagy proteins are known to be targeted by viral pathogens in order to evade or manipulate autophagic flux, it is likely that autophagy functions of TRIM proteins are targeted by specific viruses as well. Along these lines, the central role of TBK1 in IFN-mediated immunity is antagonized by a myriad of viral pathogens; however, it remains to be determined whether viral evasion strategies also target TBK1’s activity in autophagy-mediated antiviral defense. Moreover, many proteins known to play key roles in antiviral cytokine responses (including TRIMs and TBK1) have recently emerged as important regulators of autophagy. While some TRIM proteins regulate autophagy-mediated clearance of viral components, others direct the destruction of host innate immune components (such as inflammasome proteins or IRF3) via autophagy. Thus, it will be fascinating to investigate the dual roles of these molecules in both autophagy and cytokine responses during viral infection. Future discoveries in the area of autophagy-mediated host defenses by TRIM proteins may unveil new opportunities for therapeutic intervention in viral infectious diseases.
